# The U-shape relationship between insulin resistance-related indexes and chronic kidney disease: a retrospective cohort study from National Health and Nutrition Examination Survey 2007–2016

**DOI:** 10.1186/s13098-024-01408-7

**Published:** 2024-07-17

**Authors:** Ruihua Shen, Ling Lin, Zexuan Bin, Xi Qiao

**Affiliations:** 1 Department of Nephrology, Second Hospital of Shanxi Medical University, Taiyuan, People’s Republic of China; 2 Shanxi Kidney Disease Institute, Taiyuan, People’s Republic of China; 3 Kidney Research Center of Shanxi Medical University, Taiyuan, People’s Republic of China; 4 Department of Rheumatology, Second Hospital of Shanxi Medical University, Taiyuan, People’s Republic of China

**Keywords:** Chronic kidney disease, Insulin resistance, Triglyceride glucose-waist-to-height ratio, Triglyceride glucose index, National Health and Nutrition Examination Survey

## Abstract

**Background:**

There is ongoing debate on the correlation between chronic kidney disease (CKD) and insulin resistance (IR)-related indices. Our objective was to explore the prognostic ability of IR-related indexes for the prevalence of CKD, as well as the mortality from all causes and cardiovascular disease (CVD) in CKD patients.

**Methods:**

The data used in this study came from the National Health and Nutrition Examination Survey (NHANES). Binary logistic regression analysis, Cox proportional hazards model, and restricted cubic spline (RCS) were used to analyze the relationship between IR-related indexes, including metabolic score of IR (METS-IR), homeostatic model assessment for IR (HOMA-IR), triglyceride glucose index (TyG), triglyceride glucose-waist-to-height ratio (TyG-WHtR), triglyceride glucose-body mass index (TyG-BMI), with CKD and its all-cause mortality and CVD mortality. Subgroup analysis was performed to test the stability of the results. Finally, the predictive power of IR-related indexes for CKD was tested by the receiver operating characteristic (ROC) curve.

**Results:**

Among the recruited 10,660 participants, 15.42% were CKD patients. All IR-related indexes were found to be nonlinearly correlated to the prevalence of CKD in the study. When the TyG index was higher than 9.05, it was positively associated with CKD (OR: 1.77, 95% CI 1.44–2.18). Moreover, increased TyG-WHtR level was correlated with a greater prevalence of CKD when it was higher than 4.3 (OR: 1.31, 95% CI 1.19–1.45). Other IR-related indexes (METS-IR, HOMA-IR, and TyG-BMI) showed fewer notable correlations with CKD. The association of IR-related indexes and the prevalence of CKD remained consistent in most subgroups (*P* for interactions > 0.05). TyG-WHtR was also the predictor of all-cause mortality in CKD patients (HR: 1.34, 95% CI 1.14–1.58), while other IR-related indexes were not correlated with the all-cause mortality or CVD mortality in CKD patients (*P* > 0.05). Otherwise, ROC curves showed that TyG-WHtR had more robust diagnostic efficacy than other IR-related indexes (METS-IR, HOMA-IR, TyG, and TyG-BMI) in predicting CKD (area under the curve: 0.630, 95% CI 0.615–0.644).

**Conclusions:**

IR-related biomarkers (METS-IR, HOMA-IR, TyG, and TyG-BMI) were positively correlated with the prevalence of CKD. Moreover, TyG-WHtR enhanced CKD and its all-cause mortality prediction. In patients with elevated levels of IR-related indexes, the early detection and intervention of IR may reduce the occurrence of CKD and the prognosis of CKD patients.

**Supplementary Information:**

The online version contains supplementary material available at 10.1186/s13098-024-01408-7.

## Background

Chronic kidney disease (CKD) affects almost 10% of people worldwide and is linked to significant financial and public health costs [1]. One of the highest incidences of end-stage kidney disease (ESKD) in the world is still seen in the United States (US). The best management and prevention of CKD have become crucial public health concerns due to the disease's high prevalence and high cost of healthcare [2]. The weakening of the effects of insulin in skeletal muscle, adipose tissue, or liver cells is known as insulin resistance (IR), which is also defined as the state of aberrant blood glucose response linked to a specific insulin concentration [3]. In patients with CKD, the etiology of tissue insensitivity to insulin is complex and includes uremic toxins, inflammatory factors, metabolic acidosis, and renin–angiotensin–aldosterone system activation [4]. Diseases such as diabetes mellitus (DM), hypertension, and metabolic syndrome (MetS) are closely related to IR and are risk factors for CKD [5]. Based on body mass index (BMI) and MetS, CKD patients were classified into metabolically healthy normal weight/overweight/obesity and metabolically unhealthy normal weight/overweight/obesity groups according to a recent study, which demonstrated that metabolic abnormality was a significant risk factor for CKD in the Chinese population [6]. Due to its promotion of endothelial dysfunction, oxidative stress, and inflammation, IR may have a role in the onset and course of CKD [4]. Clinical studies have found that elevated IR is associated with proteinuria and CKD in patients with or without DM [7]. Therefore, IR is a serious public health issue, and early detection and management of IR can help prevent further kidney damage and improve patients' quality of life.

Hyperinsulinemic-euglycemic clamp (HEC) technique is the gold standard for assessing IR, but it has been limited in large-scale clinical trials and epidemiological investigations because of its invasiveness and low practicability [8]. Studies have shown that alternative IR markers, such as the metabolic score of IR (METS-IR) [9], homeostatic model assessment for IR (HOMA-IR) [7, 10], triglyceride glucose index (TyG) [11, 12] are closely associated with the progression of CKD. A recent study evaluated TyG-related parameters as more effective in assessing DM than isolated TyG indices, such as triglyceride glucose-waist-to-height ratio (TyG-WHtR) and triglyceride glucose-body mass index (TyG-BMI) [13]. However, the relationship between IR-related indexes and CKD is still debatable, as some research indicated that there is no significant correlation between IR-related indexes and a decline in estimated glomerular filtration rate (eGFR) in patients with or without DM [14, 15]. Prospective data concerning the association between impaired IR and mortality in CKD are scarce and conflicting. For example, research showed that IR failed to predict all-cause mortality and cardiovascular disease (CVD) mortality independently of classical risk factors [16], while another research found that TyG was a predictor of major adverse cardiovascular events (including acute myocardial infarction and ischemic stroke) in CKD patients after adjusting for demographic variables, blood lipid, blood sugar, hypertension, diabetes, smoking, and drinking [17]. Meanwhile, the effect of applying the IR-related indexes to predict the risk of CKD is unclear. Therefore, this study collected data from the National Health and Nutrition Examination Survey (NHANES) to explore the association of multiple IR-related indexes with CKD and the all-cause mortality and CVD mortality in CKD patients and the predictive value of IR-related indexes for CKD.

## Methods

### Study population

NHANES is a crucial research program that aims to assess the health and nutritional condition of both adults and children residing in the US. The national health statistics are provided by the Centers for Disease Control and Prevention (CDC), and the Research Ethics Review Board of the National Center for Health Statistics (NCHS) has formally authorized the NHANES methods. In order to safeguard the rights of the participants, NHANES has acquired informed written consent from all the individuals involved in the study. Moreover, the datasets generated and analyzed in the current study are available on the official NHANES website (https://www.cdc.gov/nchs/nhanes/index.html).

**Fig. 1:**
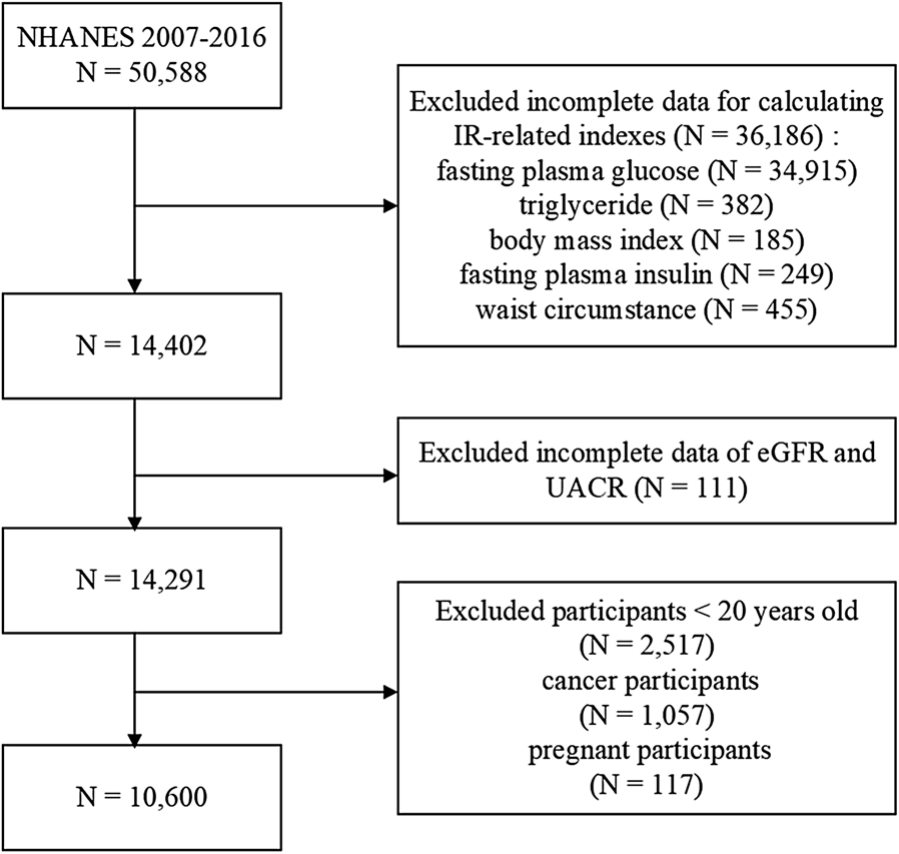
Flowchart of the sample selection from NHANES 2007–2016. *NHANES* National Health and Nutrition Examination Survey, *IR* insulin resistance, *eGFR* estimated glomerular filtration rate, *UACR* urinary albumin-to-creatinine ratio

For this study, NHANES 2007–2016 participants were obtained. After removing 36,186 patients with incomplete data for calculating the IR-related indexes [which includes fasting plasma glucose (FPG), triglyceride (TG), BMI, fasting plasma insulin, and waist circumstance], urinary albumin-to-creatinine ratio (UACR), and eGFR, as well as individuals who were younger than 20 (N = 2517), had cancer (N = 1057) or were pregnant (N = 117), we came to the conclusion that 10,660 people were suitable (Fig. 1).

### Calculation of IR-related indexes

In this study, five IR-related indexes were considered as the exposure variables. FPG, TG, and high-density lipoprotein cholesterol (HDL-C) were measured at baseline when the participants provided their blood samples. During the physical examination process, participants’ height, waist circumference, and body weight were measured in a mobile examination center. In addition, the waist-to-height ratio [WHtR (waist circumference/height)] and the BMI (body mass/height^2^) were computed. These IR-related indexes were calculated according to the following formulas:

$${\varvec{METS}}{\text{-}}{\varvec{IR}} = \frac{{\ln \left( {[2{ } \times {\text{ FPG }}\left( {{\text{mg}}/{\text{dl}}} \right) + {\text{TG }}\left( {{\text{mg}}/{\text{dl}}} \right)] \times { }\frac{{{\text{body}}\;{\text{mass}}}}{{{\text{height}}^{2} }}} \right)}}{{{\text{ln }}\left[ {{\text{HDL-C }}\left( {{\text{mg}}/{\text{dl}}} \right)} \right]}}$$

$${\varvec{HOMA}}{\text{-}}{\varvec{IR}} = \frac{{{\text{fasting}}\;{\text{plasma}}\;{\text{insulin}}\left( {{\upmu {\text{U}}}/{\text{ml}}} \right) \times {\text{FPG}}\left( {{\text{mg}}/{\text{dl}}} \right)}}{405}$$

$${\varvec{TyG}} = \frac{{{\text{ln}}\left[ {{\text{TG }}\left( {{\text{mg}}/{\text{dl}}} \right)\, \times \,{\text{FPG }}\left( {{\text{mg}}/{\text{dl}}} \right)} \right]}}{2}$$

$${\varvec{TyG}}{\text{-}}{\varvec{WHtR}} = {\text{TyG}} \times \frac{{{\text{waist}}\;{\text{circumference}}}}{{{\text{height}}}}$$

$${\varvec{TyG}}{\text{-}}{\varvec{BMI}} = {\text{TyG}} \times \frac{{{\text{body}}\;{\text{mass}}}}{{{\text{height}}^{2} }}$$

### Definition of CKD

In this study, CKD was defined as albuminuria or an eGFR of less than 60 ml/min/1.73 m^2^ [18]. Albuminuria was defined as UACR ≥ 30 mg/g [18]. As binary race classification ignores ancestral diversity within racial and ethnic groups, we used Chronic Kidney Disease Epidemiology Collaboration (CKD-EPI) without race to determine eGFR [19].

### Assessment of covariates

For demographic variables, age (categorized as 20–39, 40–59, and ≥ 60), gender (male and female), race (Mexican Hispanic, non-Hispanic white, non-Hispanic black, and other races), education levels (below high school, high school graduate, college or above), marital status (married or living with a partner and no), and poverty to income ratio (PIR) (categorized as ≤ 1.3 for low income, 1.3–3.5 for medium income, and > 3.5 for high income) were obtained from the interview by standardized questionnaires. According to the question “Do you now smoke cigarettes?”, smoking status was classified as smoking now or do not smoke now; and according to the question “Have you smoked at least 100 cigarettes in your entire life?”, the participants who did not smoke now were divided into having a smoking history or never smoking. According to the question “Had at least 12 alcohol drinks/lifetime?”, alcohol status was divided into yes or no. BMI was categorized into three groups: < 25, 25 to 29.9, and ≥ 30  kg/m^2^. Hypertension was defined as the mean blood pressure value of three measurements ≥ 130 mmHg for systolic blood pressure (SBP) or ≥ 80 mmHg for diastolic blood pressure (DBP), or having been informed by a physician of a diagnosis of hypertension [20]. Prediabetes is identified by having 100 ≤ FBG ≤ 125 mg/dl, 140 mg/dl ≤ oral glucose tolerance test (OGTT) ≤ 199 mg/dl, or 5.7% ≤ hemoglobin A1c (HbA1c) < 6.5% [21]. Diabetes was defined by self-reported diagnosis, use of insulin or oral hypoglycemic medication, FBG ≥ 126 mg/dl, OGTT ≥ 200 mg/dl, or HbA1c ≥ 6.5% [21]. Hyperlipidemia was defined as having total cholesterol ≥ 200 mg/dl, TG ≥ 150 mg/dl, HDL-C < 40 mg/dl in males and < 50 mg/dl in females, or low-density lipoprotein cholesterol ≥ 130 mg/dl [22]. Hyperuricemia was delineated as a serum uric acid ≥ 7.0 mg/dl in males or ≥ 6.0 mg/dl in females [23]. MetS was characterized by the following diagnostic criteria, requiring three of five factors [22]: (1) TG ≥ 150 mg/dL; (2) HDL-C < 40 mg/dl in male or < 50 mg/dl in female; (3) FPG ≥ 100 mg/dl; (4) waist circumference > 102 cm in male or > 88 cm in female; (5) SBP ≥ 130 mmHg and/or DBP ≥ 85 mmHg.

### Statistical analysis

Categorical variables were presented as counts with percentages, and the Chi-square test or Fisher’s exact probability test (counting variables with theoretical numbers < 10) was performed for statistical analysis. Continuous variables were presented as mean values with standard deviations (SD), Kruskal–Wallis test was used to assess the differences between groups.

We considered IR-related indexes as continuous variables and then divided the total participants into four quartiles for further analysis. Next, three logistic regression models were used to examine the association of the quartiles of IR-related indexes with CKD. The lowest quartile (Q1) was a reference, then odd ratios (ORs) along with their 95% confidence interval (CI) for Quartile 2 (Q2), Quartile 3 (Q3), and Quartile 4 (Q4) were calculated. The Cox proportional hazards model was used to estimate hazard ratios (HRs) and 95% CI for the association between IR-related indexes and all-cause mortality and cardiovascular disease (CVD) mortality. Model 1 was unadjusted; model 2 was adjusted for age, gender, race, education level, marital status, and PIR; and model 3 was further adjusted for BMI, smoking status, alcohol status, hypertension, abnormal glucose metabolism, hyperlipidemia, hyperuricemia.

The dose–response relationship (linear or nonlinear) between IR-related indexes and CKD, or the all-cause mortality and CVD mortality of CKD patients was investigated by the restricted cubic spline (RCS) model. Models fitted by RCS were adjusted for the same covariates as in model 3 performed.

Subgroup analysis was conducted to assess potential moderating effects of age (20–39, 40–59, and ≥ 60), gender (male and female), race (Mexican Hispanic, non-Hispanic white, non-Hispanic black, and other races), education level (below high school, high school graduate, college or above), marital status (married or living with a partner and no), PIR (low income, medium income, and high income), BMI (< 25, 25–29.9, and ≥ 30), smoking status (never, smoking history, and smoking now), alcohol status (yes and no), hypertension (yes and no), abnormal glucose metabolism (diabetes, prediabetes, and no), hyperlipidemia (yes and no), and hyperuricemia (yes and no).

Receiver operating characteristic (ROC) curves were used for diagnostic value analysis, and the area under the curve (AUC) was calculated to quantify the predictive power of IR-related indexes for CKD.

All analysis was performed with STATA version 15.0 and R version 4.3.0. A two-sided *P* value < 0.05 was considered statistically significant.

## Results

### Participants’ characteristics at baseline

Among the 10,660 participants in our analysis, 15.42% of whom were CKD patients. Women made up 50.31% and people over 60 made up 29.00% of all participants. The proportion of Mexican Americans was 16.41%. As shown in Table 1, compared to non-CKD individuals, those CKD patients were more likely to be female, older, non-married or living with a partner, smoker, drinker, to have lower levels of educational strata, lower income, higher BMI, BP, blood glucose, blood lipids, and blood uric acid. Meanwhile, as compared to non-CKD participants, all IR-related indexes were notably higher in CKD patients (*P* < 0.05).

**Table 1:** Baseline variables according to the CKD group

	Non-CKD	CKD	*P* value
N	8956	1644	
Gender, n (%)			0.031
Female	4491 (50.15%)	872 (53.04%)	
Male	4465 (49.85%)	772 (46.96%)	
Age, n (%)			< 0.001
20–39	3420 (38.19%)	296 (18.00%)	
40–59	3329 (37.17%)	464 (28.22%)	
≥ 60	2207 (24.64%)	884 (53.77%)	
Race, n (%)			< 0.001
Mexican American	1477 (16.49%)	272 (16.55%)	
Non-Hispanic White	3609 (40.30%)	662 (40.27%)	
Non-Hispanic Black	1722 (19.23%)	379 (23.05%)	
Others	2148 (23.98%)	331 (20.13%)	
Education level, n (%)			< 0.001
Below high school	2205 (24.62%)	581 (35.34%)	
High school graduate	1940 (21.66%)	408 (24.82%)	
College or above	4804 (53.64%)	652 (39.66%)	
Missing	7 (0.08%)	3 (0.18%)	
Marital status, n (%)			< 0.001
Married or living with a partner	5496 (61.37%)	895 (54.44%)	
No	3460 (38.63%)	749 (45.56%)	
PIR, n (%)			< 0.001
Low income	2695 (30.09%)	578 (35.16%)	
Medium income	2978 (33.25%)	613 (37.29%)	
High income	3283 (36.66%)	453 (27.55%)	
BMI, n (%)			< 0.001
< 25	2697 (30.11%)	432 (26.28%)	
25–29.9	3077 (34.36%)	474 (28.83%)	
≥ 30	3182 (35.53%)	738 (44.89%)	
Smoking status, n (%)			< 0.001
Smoking now	1500 (16.75%)	299 (18.19%)	
Smoking history	2358 (26.33%)	499 (30.35%)	
Never	5098 (56.92%)	846 (51.46%)	
Alcohol status, n (%)			0.006
Yes	1062 (11.86%)	235 (14.29%)	
No	7894 (88.14%)	1409 (85.71%)	
Hypertension, n (%)			< 0.001
Yes	4053 (45.25%)	1226 (74.57%)	
No	4903 (54.75%)	418 (25.43%)	
Abnormal glucose metabolism, n (%)			< 0.001
Diabetes	1399 (15.62%)	703 (42.76%)	
Prediabetes	2790 (31.15%)	502 (30.54%)	
No	4767 (53.23%)	439 (26.70%)	
Hyperlipidemia, n (%)			< 0.001
Yes	5588 (62.39%)	1098 (66.79%)	
No	3368 (37.61%)	546 (33.21%)	
Hyperuricemia, n (%)			< 0.001
Yes	1617 (18.05%)	560 (34.06%)	
No	7339 (81.95%)	1084 (65.94%)	
MetS, n (%)			< 0.001
Yes	2449 (27.34%)	827 (50.30%)	
No	6507 (72.66%)	817 (49.70%)	
METS-IR, mean ± SD	42.64 ± 11.95	45.92 ± 13.91	< 0.001
HOMA-IR, mean ± SD	3.61 ± 4.84	5.78 ± 11.74	< 0.001
TyG, mean ± SD	8.57 ± 0.66	8.86 ± 0.78	< 0.001
TyG-WHtR, mean ± SD	5.04 ± 1.01	5.54 ± 1.13	< 0.001
TyG-BMI, mean ± SD	247.50 ± 63.52	266.67 ± 73.86	< 0.001

Continuous variables were listed as mean ± standard deviation (SD), and the Kruskal–Wallis test was conducted to compare continuous baseline characteristics. Categorical variables were listed as counts and percentages, and Chi-square tests or Fisher’s exact probability test was conducted to compare categorical baseline characteristics

*CKD* chronic kidney disease, *PIR* poverty to income ratio, *BMI* body mass index, *MetS* metabolic syndrome, *METS-IR* metabolic score for insulin resistance, *HOMA-IR* homeostatic model assessment for insulin resistance, *TyG* triglyceride glucose index, *TyG-WHtR* triglyceride glucose-waist-to-height ratio, *TyG-BMI* triglyceride glucose-body mass index

### Associations of IR-related indexes with CKD

The associations between IR-related indexes and CKD are shown in Tables 2, 3, 4, 5, 6. The results revealed a positive association between IR-related indexes (continuous) and CKD with statistical significance (*P* < 0.05). After confounders adjusted, TyG had the highest correlation with CKD (OR: 1.30, 95% CI 1.17–1.44), followed by TyG-WHtR (OR: 1.27, 95% CI 1.16–1.39), HOMA-IR (OR: 1.02, 95% CI 1.01–1.02), METS-IR (OR: 1.01, 95% CI 1.00–1.02), TyG-BMI (OR: 1.00, 95% CI 1.00–1.00). The risk of CKD was significantly higher with TyG-WHtR Q3 (OR: 1.44, 95% CI 1.12–1.86) and Q4 (OR: 1.82, 95% CI 1.36–2.44) compared to TyG-WHtR Q1 (reference group), with *P* for trend < 0.0001. However, after being grouped as quartiles, there was no significant correlation between other IR-related indexes (METS-IR, HOMA-IR, TyG, and TyG-BMI) and CKD in model 3.

**Table 2:** Associations of metabolic score of insulin resistance (METS-IR) with the risk of chronic kidney disease

	METS-IR continuous OR (95% CI)	METS-IR quantiles OR (95% CI)				*P* for trend
		Q1	Q2	Q3	Q4	
Model 1^a^	1.02 (1.02, 1.02)	Reference	1.02 (0.87, 1.19)	1.22 (1.04, 1.42)	1.79 (1.54, 2.07)	< 0.0001
Model 2^b^	1.02 (1.02, 1.03)	Reference	0.86 (0.73, 1.02)	1.00 (0.85, 1.17)	1.62 (1.39, 1.89)	< 0.0001
Model 3^c^	1.01 (1.00, 1.02)	Reference	0.88 (0.70, 1.10)	0.84 (0.63, 1.11)	0.99 (0.71, 1.37)	0.025

*OR* odd ratio, *95% CI* 95% confidence interval

^a^Model 1 was unadjusted

^b^Model 2 was adjusted for age, gender, race, education level, marital status, and poverty to income ratio

^c^Model 3 includes adjustment for variables in model 2 plus body mass index, smoking status, alcohol status, hypertension, abnormal glucose metabolism, hyperlipidemia, and hyperuricemia

**Table 3:** Associations of homeostatic model assessment for insulin resistance (HOMA-IR) with the risk of chronic kidney disease

	HOMA-IR continuous OR (95% CI)	HOMA-IR quantiles OR (95% CI)				*P* for trend
		Q1	Q2	Q3	Q4	
Model 1^a^	1.04 (1.04, 1.05)	Reference	1.04 (0.88, 1.22)	1.14 (0.97, 1.33)	1.95 (1.68, 2.26)	< 0.0001
Model 2^b^	1.04 (1.03, 1.05)	Reference	0.93 (0.79, 1.10)	0.99 (0.84, 1.17)	1.65 (1.42, 1.93)	< 0.0001
Model 3^c^	1.02 (1.01, 1.02)	Reference	0.87 (0.73, 1.04)	0.79 (0.65, 0.95)	0.90 (0.74, 1.09)	< 0.0001

*OR* odd ratio, *95% CI* 95% confidence interval

^a^Model 1 was unadjusted

^b^Model 2 was adjusted for age, gender, race, education level, marital status, and poverty to income ratio

^c^Model 3 includes adjustment for variables in model 2 plus body mass index, smoking status, alcohol status, hypertension, abnormal glucose metabolism, hyperlipidemia, and hyperuricemia

**Table 4:** Associations of triglyceride glucose index (TyG) with the risk of chronic kidney disease

	TyG continuous OR (95% CI)	TyG quantiles OR (95% CI)				*P* for trend
		Q1	Q2	Q3	Q4	
Model 1^a^	1.78 (1.65, 1.91)	Reference	1.27 (1.07, 1.50)	1.62 (1.37, 1.90)	2.57 (2.21, 3.00)	< 0.0001
Model 2^b^	1.70 (1.57, 1.85)	Reference	1.09 (0.91, 1.29)	1.33 (1.12, 1.58)	2.09 (1.77, 2.47)	< 0.0001
Model 3^c^	1.30 (1.17, 1.44)	Reference	0.95 (0.79, 1.14)	1.03 (0.85, 1.24)	1.21 (0.99, 1.49)	< 0.0001

*OR* odd ratio, *95% CI* 95% confidence interval

^a^Model 1 was unadjusted

^b^Model 2 was adjusted for age, gender, race, education level, marital status, and poverty to income ratio

^c^Model 3 includes adjustment for variables in model 2 plus body mass index, smoking status, alcohol status, hypertension, abnormal glucose metabolism, hyperlipidemia, and hyperuricemia

**Table 5:** Associations of triglyceride glucose-waist-to-height ratio (TyG-WHtR) with the risk of chronic kidney disease

	TyG-WHtR continuous OR (95% CI)	TyG-WHtR quantiles OR (95% CI)				*P* for trend
		Q1	Q2	Q3	Q4	
Model 1^a^	1.55 (1.47, 1.62)	Reference	1.48 (1.24, 1.77)	1.90 (1.60, 2.25)	3.34 (2.84, 3.91)	< 0.0001
Model 2^b^	1.40 (1.32, 1.47)	Reference	1.13 (0.94, 1.35)	1.34 (1.12, 1.61)	2.20 (1.86, 2.61)	< 0.0001
Model 3^c^	1.27 (1.16, 1.39)	Reference	1.22 (0.98, 1.50)	1.44 (1.12, 1.86)	1.82 (1.36, 2.44)	< 0.0001

*OR* odd ratio, *95% CI* 95% confidence interval

^a^Model 1 was unadjusted

^b^Model 2 was adjusted for age, gender, race, education level, marital status, and poverty to income ratio

^c^Model 3 includes adjustment for variables in model 2 plus body mass index, smoking status, alcohol status, hypertension, abnormal glucose metabolism, hyperlipidemia, and hyperuricemia

**Table 6:** Associations of triglyceride glucose-body mass index (TyG-BMI) with the risk of chronic kidney disease

	TyG-BMI continuous OR (95% CI)	TyG-BMI quantiles OR (95% CI)				*P* for trend
		Q1	Q2	Q3	Q4	
Model 1^a^	1.00 (1.00, 1.01)	Reference	1.12 (0.95, 1.31)	1.24 (1.06, 1.45)	1.94 (1.67, 2.25)	< 0.0001
Model 2^b^	1.00 (1.00, 1.00)	Reference	0.90 (0.76, 1.06)	0.98 (0.83, 1.16)	1.62 (1.38, 1.89)	< 0.0001
Model 3^c^	1.00 (1.00, 1.00)	Reference	0.90 (0.72, 1.14)	0.81 (0.60, 1.10)	0.92 (0.65, 1.32)	0.048

*OR* odd ratio, *95% CI* 95% confidence interval

^a^Model 1 was unadjusted

^b^Model 2 was adjusted for age, gender, race, education level, marital status, and poverty to income ratio

^c^Model 3 includes adjustment for variables in model 2 plus body mass index, smoking status, alcohol status, hypertension, abnormal glucose metabolism, hyperlipidemia, and hyperuricemia

### RCS analysis investigating the relationship between IR-related indexes and CKD

**Fig. 2:**
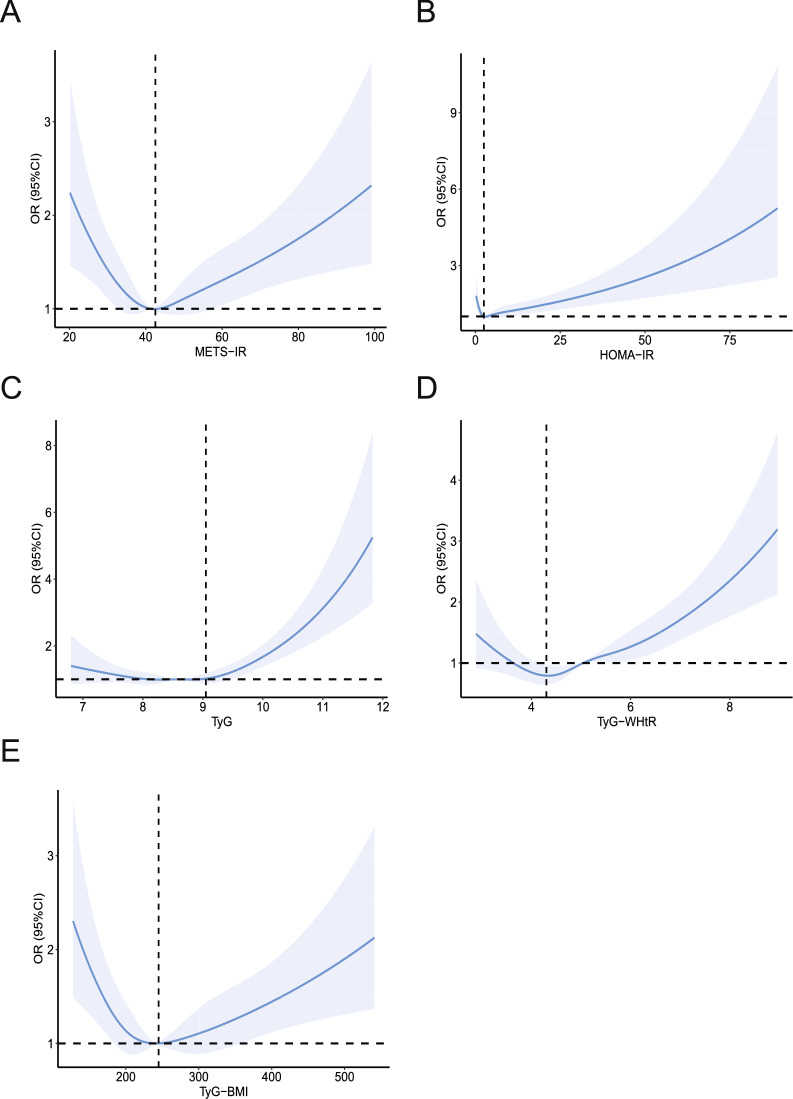
Associations between **A** metabolic score of insulin resistance (METS-IR), **B** homeostatic model assessment for insulin resistance (HOMA-IR), **C** triglyceride glucose index (TyG), **D** triglyceride glucose-waist-to-height ratio (TyG-WHtR), and **E** triglyceride glucose-body mass index (TyG-BMI) with the risk of chronic kidney disease were evaluated by restricted cubic spline after adjustment for the covariables in model 3. The solid blue lines correspond to the central estimates, and the light blue regions indicate the 95% confidence intervals. The dashed lines parallel to the X-axis indicate that odd ratio = 1, and the dashed lines parallel to the Y-axis indicate that the X value is equal to the turning point

As the logistic regression models, which classified the IR-related indexes as quartiles, did not reveal any difference after the confounders were adjusted, we employed RCS to model and illustrate the non-linear association between IR-related measures and CKD in a flexible manner. The findings revealed a U-shape relationship between CKD participants and the IR-related indicators (Fig. 2). After confounders adjusted, the risk of CKD increases in model 3 when the METS-IR index is greater than 42.5 (*P*-overall < 0.0001 and *P*-nonlinear < 0.0001) (Fig. 2A), the HOMA-IR index is greater than 2.5 (*P*-overall < 0.0001 and *P*-nonlinear = 0.0003) (Fig. 2B), the TyG index is greater than 9.05 (*P*-overall < 0.0001 and *P*-nonlinear < 0.0001) (Fig. 2C), the TyG-WHtR index is greater than 4.3 (*P*-overall < 0.0001 and *P*-nonlinear = 0.0006) (Fig. 2D), or when the TyG-BMI index is greater than 245 (*P*-overall < 0.0001 and *P*-nonlinear < 0.0001) (Fig. 2E). Further research was done using the logistic regression model based on the turning points of IR-related indexes (Table 7). We discovered statistically significant positive correlations between CKD and IR-related indices (*P* < 0.05). TyG had the highest positive correlation with the prevalence of CKD (OR: 1.77, 95% CI 1.44–2.18, followed by TyG-WHtR (OR: 1.31, 95% CI 1.19–1.45), HOMA-IR (OR: 1.02, 95% CI 1.01–1.03), METS-IR (OR: 1.01, 95% CI 1.01–1.02), TyG-BMI (OR: 1.00, 95% CI 1.00–1.00).

**Table 7:** Associations of insulin resistance**-**related indexes with the risk of chronic kidney disease after turning points

	METS-IR (≥ 42.5) OR (95% CI)	HOMA-IR (≥ 2.5) OR (95% CI)	TyG (≥ 9.05) OR (95% CI)	TyG-WHtR (≥ 4.3) OR (95% CI)	TyG-BMI (≥ 245) OR (95% CI)
	N = 4,881	N = 5,396	N = 2,466	N = 8,146	N = 5,062
Model 1^a^	1.02 (1.02, 1.03)	1.04 (1.03, 1.05)	2.00 (1.67, 2.39)	1.57 (1.47, 1.67)	1.00 (1.00, 1.01)
Model 2^b^	1.03 (1.02, 1.04)	1.04 (1.03, 1.05)	2.22 (1.84, 2.69)	1.49 (1.40, 1.60)	1.01 (1.00, 1.01)
Model 3^c^	1.01 (1.01, 1.02)	1.02 (1.01, 1.03)	1.77 (1.44, 2.18)	1.31 (1.19, 1.45)	1.00 (1.00, 1.00)

*METS-IR* metabolic score for insulin resistance, *HOMA-IR* homeostatic model assessment for insulin resistance, *TyG* triglyceride glucose index, *TyG-WHtR* triglyceride glucose-waist-to-height ratio, *TyG-BMI* triglyceride glucose-body mass index, *OR* odd ratio, *95% CI* 95% confidence interval

^a^Model 1 was unadjusted

^b^Model 2 was adjusted for age, gender, race, education level, marital status, and poverty to income ratio

^c^Model 3 includes adjustment for variables in model 2 plus body mass index, smoking status, alcohol status, hypertension, abnormal glucose metabolism, hyperlipidemia, and hyperuricemia

### Subgroup analysis of the correlation between the IR-related indexes and CKD

**Fig. 3:**
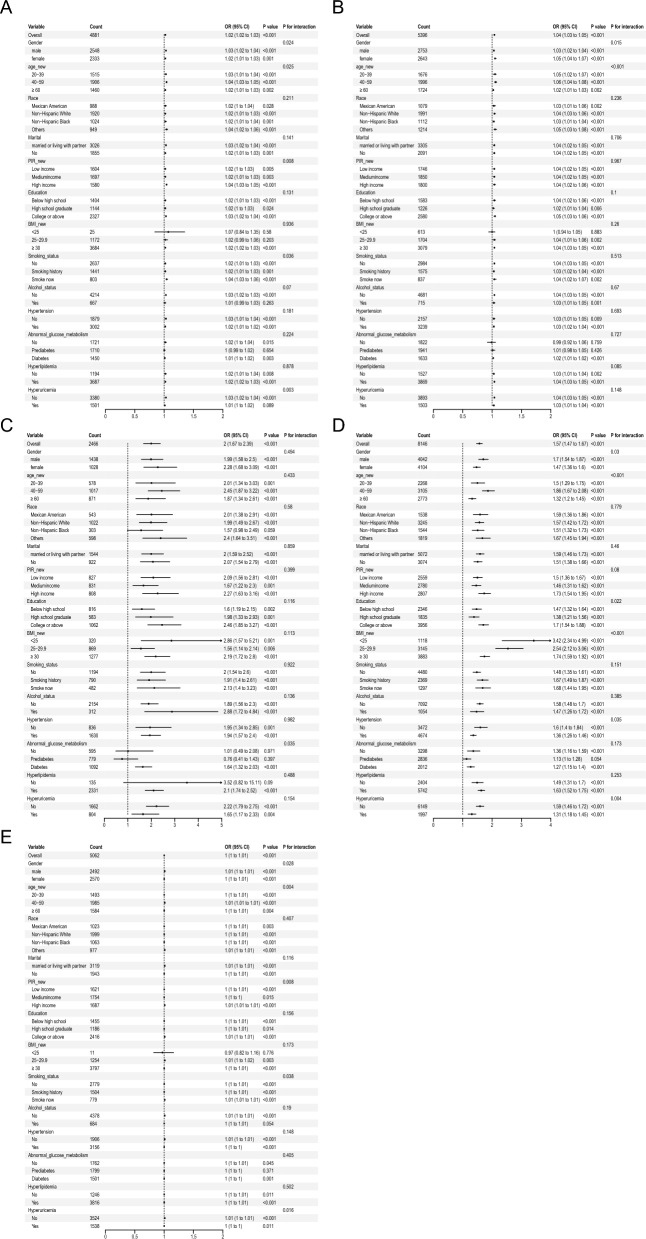
Forest plot of **A** metabolic score of insulin resistance (METS-IR), **B** homeostatic model assessment for insulin resistance (HOMA-IR), **C** triglyceride glucose index (TyG), **D** triglyceride glucose-waist-to-height ratio (TyG-WHtR), and **E** triglyceride glucose-body mass index (TyG-BMI) association with the risk of chronic kidney disease

The association of 5 surrogate markers of IR and the prevalence of CKD remained consistent in most subgroups (*P* for interactions > 0.05). Nevertheless, with METS-IR ≥ 42.5, there was a strong positive correlation between the METS-IR and the prevalence of CKD in participants who were male, 40–59 years old, smokers, and those who had high income and without hyperuricemia (Fig. 3A). Additionally, there was a significant interaction between subgroups stratified by gender and age between HOMA-IR index and the prevalence of CKD, female and 40–59 years old participants were more likely to have CKD when HOMA-IR ≥ 2.5 (Fig. 3B). The TyG index and the prevalence of CKD showed a more positive relationship in individuals with diabetes when TyG was greater than 9.05 (Fig. 3C). In addition, the following variables were associated with a higher risk of CKD when TyG-WHtR ≥ 4.3, the risk of CKD was higher with the following characteristics: male, 40–59 years old, high education level, lower BMI, and absence of hypertension or hyperuricemia (Fig. 3D). The following factors increased the risk of CKD when TyG-BMI ≥ 245: male, age 40–59, higher income, smokers, and absence of hyperuricemia (Fig. 3E).

### Diagnostic efficacy of IR-related indexes for CKD

**Fig. 4:**
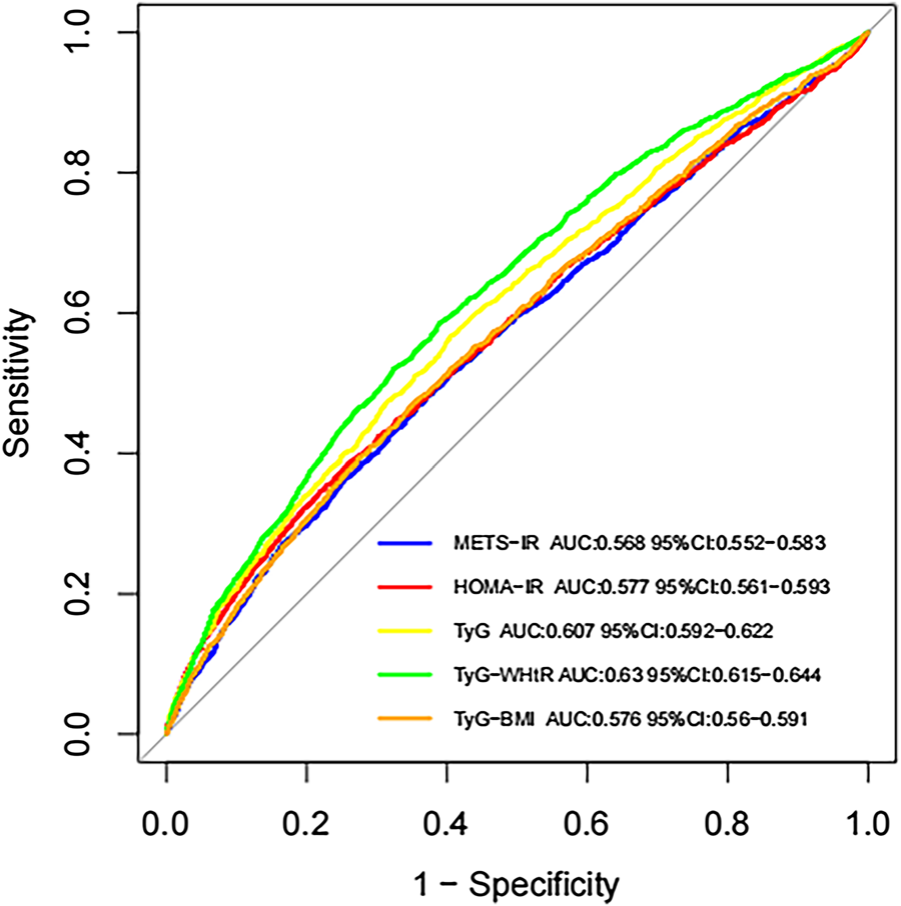
Diagnostic efficacy of metabolic score of insulin resistance (METS-IR), homeostatic model assessment for insulin resistance (HOMA-IR), triglyceride glucose index (TyG), triglyceride glucose-waist-to-height ratio (TyG-WHtR), and triglyceride glucose-body mass index (TyG-BMI) for chronic kidney disease

The receiver operating characteristic (ROC) curve was used to analyze the diagnostic efficacy of the IR-related indexes for CKD (Fig. 4). An AUC greater than 0.5 is considered to have diagnostic applications, TyG-WHtR was the best predictive index (AUC: 0.630, 95% CI 0.615–0.644), followed by TyG (AUC: 0.607, 95% CI 0.592–0.622), HOMA-IR (AUC: 0.577, 95% CI 0.561–0.593), TyG-BMI (AUC: 0.576, 95% CI 0.560–0.591), and METS-IR (AUC: 0.568, 95% CI 0.552–0.583).

### Associations of IR-related indexes with all-cause mortality and CVD mortality in CKD patients

**Fig. 5:**
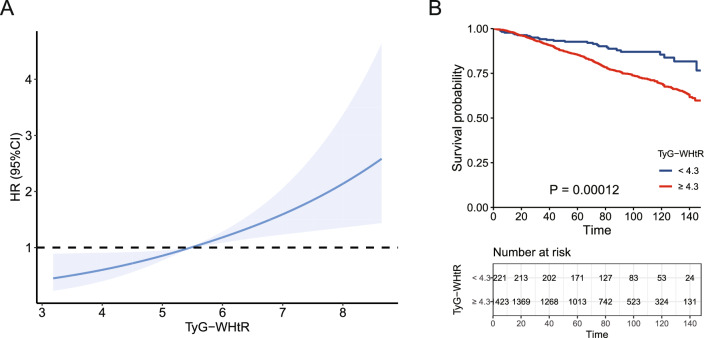
**A** Associations between triglyceride glucose-waist-to-height ratio (TyG-WHtR) and all-cause mortality in chronic kidney disease (CKD) patients. **B** The Kaplan–Meier curve for all-cause mortality of CKD patients based on different TyG-WHtR

Supplementary tables provide detailed information on all associations of IR-related indexes with all-cause mortality and CVD mortality in CKD patients. After adjustment for covariates, the results demonstrated that TyG-WHtR was positively correlated with all-cause mortality in CKD patients (HR: 1.34, 95% CI 1.14–1.58) (Table 8), and the RCS curve revealed a linear correlation between TyG-WHtR and the all-cause mortality of CKD patients (*P*-overall = 0.0005, *P*-nonlinear = 0.7834) (Fig. 5A). Nevertheless, there was no discernible relationship between TyG-WHtR and CKD patients’ CVD mortality (Table 8). Meanwhile, METS-IR, HOMA-IR, TyG, and TyG-BMI were not significantly associated with all-cause mortality or CVD mortality in CKD patients (supplementary Tables 1–4). After adjusting for all covariates in model 3 above, the Kaplan–Meier curve showed that the cumulative probability of survival was reduced in CKD patients with TyG-WHtR ≥ 4.3 compared to those with TyG-WHtR < 4.3 (Fig. 5B).

**Table 8:** Association of triglyceride glucose-waist-to-height ratio (TyG-WHtR) with all-cause mortality and cardiovascular disease (CVD) mortality in chronic kidney disease patients

	TyG-WHtR HR (95% CI)	*P* value
All-cause mortality		
Model 1^a^	1.11 (1.01, 1.21)	0.022
Model 2^b^	1.05 (0.95, 1.16)	0.336
Model 3^c^	1.34 (1.14, 1.58)	0.0005
CVD mortality		
Model 1^a^	1.06 (0.90, 1.24)	0.516
Model 2^b^	1.07 (0.91, 1.26)	0.425
Model 3^c^	1.10 (0.82, 1.46)	0.527

*HR* hazard ratio, *95% CI* 95% confidence interval

^a^Model 1 was unadjusted

^b^Model 2 was adjusted for age, gender, race, education level, marital status, and poverty to income ratio

^c^Model 3 includes adjustment for variables in model 2 plus body mass index, smoking status, alcohol status, hypertension, abnormal glucose metabolism, hyperlipidemia, and hyperuricemia

## Discussion

This cross-sectional study conducted on 10,660 adult participants revealed a U-shape relationship between the occurrence of CKD and IR-related indexes. Specifically, an increase in IR-related indices was positively associated with a higher prevalence of CKD, particularly when the following thresholds were met: METS-IR ≥ 42.5, HOMA-IR ≥ 2.5, TyG ≥ 9.05, TyG-WHtR ≥ 4.3, and TyG-BMI ≥ 245. Subgroup studies and interaction tests demonstrated that different groups with elevated IR-related indices had distinct associations with CKD risk. ROC analysis revealed that TyG-WHtR might serve as a more reliable predictor of CKD compared to other IR-related indices, including METS-IR, HOMA-IR, TyG, and TyG-BMI. Additionally, TyG-WHtR was positively linked to the all-cause mortality rates among CKD patients. Lastly, it's critical to stress and emphasize the importance of high IR levels when evaluating adult Americans’ renal health.

One of the main effects of IR is hyperinsulinemia, which impairs kidney function by increased vascular permeability, endothelial dysfunction, and glomerular hyperfiltration [4]. In a longitudinal study involving patients with CKD, individuals who exhibited IR were observed to have a greater decline in renal function compared to insulin-sensitive people [24]. However, another study indicated that in non-diabetic CKD patients with eGFR between 20–70 ml/min/1.73 m^2^, HOMA-IR is not associated with ESKD or eGFR reduction (HR: 1.01, 95% CI 0.90–1.14) [14]. Meanwhile, there was no significant correlation between TyG index and type 2 diabetes mellitus (T2DM) patients whose eGFR was less than 30 ml/min/1.73 m^2^) [15]. Variations in study outcomes, sample size, design, and statistical techniques could be the cause of the discrepancies in these results. It is important to note that the above studies mainly focused on hospitalized patients, so further validation in the community population is required.

It was demonstrated that METS-IR, a potentially useful method for IR screening, was highly compatible with the outcomes of HEC [25]. Recent studies of the Chinese population have established a connection between a higher METS-IR and an elevated risk of several renal outcomes, including higher UACR, a rapid decline in eGFR, and a mild reduction in eGFR [26, 27]. Previous research has demonstrated that there are population-specific differences in the relationship between METS-IR and the risk of eGFR decrease or UACR increase. Among smokers and individuals without hyperuricemia, METS-IR was adversely correlated with eGFR [9]. A Chinese study showed that the positive correlation between MEST-IR and UACR was more significant in men [26]. According to a new meta-analysis, current and past smokers had far higher risks of CKD than non-smokers (OR: 1.18, 95% CI 1.10–1.27) [28]. Consistent with the previous findings, the subgroup analysis conducted in this study also demonstrated a significant association between elevated METS-IR and the risk of CKD among males, smokers, and individuals without hyperuricemia. Additionally, male individuals aged 40–59 and those with high incomes showed a stronger correlation between METS-IR and the risk of CKD. This finding could be explained by the protective effect of estrogen, elevated pressure from life and work throughout this age range, and dietary differences resulting from family income.

HOMA-IR, combining fasting insulin and fasting glucose, was used to measure IR. It was shown that HOMA-IR exhibited a robust correlation with CKD in Korean adults, regardless of their T2DM status [7]. A comparable Iranian cohort study revealed that a one-unit increase in HOMA-IR was associated with a 72% and 37% increased risk of CKD in males and females, respectively [29]. In another study conducted among Chinese patients with MetS, a positive association was observed between HOMA-IR and the prevalence of CKD, but it was not statistically significant in females (male OR: 1.21, 95% CI 1.14–1.28, *P* ≤ 0.001; female OR: 1.01, 95% CI 0.99–1.02, *P* = 0.38) [30]. However, the subgroup analysis in this study revealed that the association between an elevated HOMA-IR and the risk of CKD was notably stronger among female participants compared to males, which may be attributed to the difference in the cross-sectional study design and patient categories that were collected. Similar to METS-IR, there was a stronger correlation between increased HOMA-IR and the risk of CKD in the 40–59 age group than in the ≥ 60 age group. This could potentially be explained by the greater work and life stress experienced by this age group, and the association between the two needs to be further explored. A recent study on the Korean population showed that METS-IR was more predictable than HOMA-IR in the prediction of incident albuminuria [31]. Our research results showed that there was no significant difference in the prediction of CKD between METS-IR and HOMA-IR, which may be caused by the differences in the included population.

A prospective cohort study with a median follow-up of 17.5 years demonstrated a positive correlation between the TyG index and the risk of CKD [24]. Furthermore, a cohort study in China found that the relationship between TyG and CKD was non-linear (*P*-nonlinear = 0.021). When TyG ≥ 8.94, the risk of CKD increased rapidly with the increase of TyG [12]. In line with previous investigations, the RCS curve results of this study also indicated a non-linear association between TyG and CKD, with a turning point of approximately 9.05 for TyG. In addition, a recent study has established a link between TyG levels and the risk of CKD progression among patients with CKD and DM [32], and similar findings were observed in this study’s subgroup analysis. Another clinical study showed that TyG can also independently predict the occurrence of CKD in non-diabetic people [33], while our study found that there was no significant correlation between TyG and CKD risk in non-diabetic participants, which might be a result of different study designs and uncontrolled confounding bias. A recent study on the Korean population showed that TyG was more predictable in the prediction of incident albuminuria than HOMA-IR [31], and we also found similar results.

The TyG combined obesity index, including both TyG-WHtR and TyG-BMI, serves as a more accurate indicator of DM compared to the TyG index alone [13]. A linear regression analysis conducted on Chinese individuals aged 45 and above revealed that TyG-BMI was significantly positively correlated with serum creatinine and negatively correlated with eGFR [34]. Another study included CKD stages 1–4 patients, indicating a negative correlation between TyG-BMI and unfavorable renal outcomes. Specifically, patients with TyG-BMI in the Q1 range (HR: 1.86, 95% CI 1.19–2.91) and Q2 range (HR: 1.57, 95% CI 1.10–2.23) exhibited a significantly elevated risk of adverse renal outcomes when compared to those in the Q4 group, which served as the reference category [35]. There was no clinical research that connected TyG-WHtR to the risk of proteinuria, CKD, or eGFR decrease. The results of this study suggested that TyG-WHtR and TyG-BMI are U-shapely correlated with the risk of CKD, and the risk of CKD rises with the above index when the threshold is crossed. Moreover, the stratified analysis showed that the correlation between TyG-WHtR or TyG-BMI and the risk of CKD varied among different population groups. Similar to METS-IR, TyG-WHtR, and TyG-BMI exhibited stronger positive associations with CKD in the non-hyperuricemia population. However, there is currently a lack of cohort studies exploring the correlation between TyG-WHtR and TyG-BMI and the risk of renal function progression in hyperuricemia patients, and the correlation between the two needs to be further explored.

Previous studies have not evaluated the nonlinear relationship between multiple IR-related indices and CKD, and our study fills these gaps. A previous study demonstrated that prior episodes of severe hypoglycemia were linked to an elevated risk of ESKD in T2DM patients [36]. All IR-related indexes involved in this study include FPG, consequently, low FPG may be the cause of the negative correlation found between abnormally low IR-related indicators and an elevated risk of CKD. Malnutrition has been linked to increased morbidity, decreased functional ability, and an increase in the frequency and length of hospital hospitalizations [37]. BMI offers important information for evaluating nutritional status. Low BMI is a common symptom of poor nutritional and calorie intake in CKD patients, which negatively impacts muscle protein synthesis and metabolism [38]. WHtR is a measure of central obesity, and a U-shape association was found between BMI or WHtR and UACR and microalbuminuria [39]. Therefore, low nutritional status may be the cause of the negative connection between very low IR-related indicators and an elevated risk of CKD. Cross-sectional epidemiological studies are often not appropriate for causality and pathophysiology estimation. As a result, more research is required to fully understand the U-shape relationship occurrence between IR-related indexes and CKD, particularly in animal studies or intervention trials.

According to the results of the Cox proportional hazards model, we found a positive linear relationship between TyG-WHtR and CKD patients’ all-cause mortality, while a significant correlation between other IR-related indexes and the all-cause mortality and CVD mortality of CKD patients was not observed. A recent study showed that TyG, TyG-BMI, and METS-IR were not associated with all-cause mortality in stages 1–4 CKD patients [35], which was in line with the results of our study. However, a retrospective cohort study of the Chinese population found that TyG was a new predictor of major adverse cardiovascular events (including acute myocardial infarction and ischemic stroke) in CKD patients [17]. Another study revealed that IR assessed by TyG-BMI was independently associated with an increased risk of CVD mortality and all-cause mortality in patients with peritoneal dialysis [40]. The inconsistent results might be partially explained by different methods for quantification of IR, and more experimental and clinical research is therefore required in order to clarify the prognostic implications of IR in CKD.

The study has several advantages that set it apart from previous studies. First, based on population-based sample survey data, this study reports results from real-world clinical practice that reflect real-world conditions. Notably, it demonstrated significant associations between IR-related indexes and CKD even after adjusting confounding variables, indicating that the IR-correlated indexes have the potential to be useful, practical, and direct measures of CKD treatment and management. Second, we also conducted a subgroup analysis stratified by different confounders and identified associations between IR-related indexes and CKD in different populations. For example, individuals aged 40–59 years old, who smoke, without hyperuricemia were shown to have an increased risk of CKD when IR-related indexes were elevated. Therefore, in clinical settings, clinicians should focus more on IR and kidney conditions in these populations. Nevertheless, future studies should aim to establish a safe threshold for IR-related indexes to guide pharmacological therapy in patients with CKD, as the complexity of the disease and the presence of numerous combined risk factors in patients with CKD may lessen the relationship between IR-related indexes and CKD.

Some potential limitations are also worth noting. First, the results of this study were based on the calculation of IR-related indicators at baseline, and the cross-sectional study design restricted the inference of causality. Further prospective studies and longitudinal cohort studies with multiple IR-related index calculations are needed to further explore the relationship between IR-related indexes and CKD [7]. Second, the data in this study came from the US population, so it is uncertain whether the conclusions obtained can be applied to populations in other regions. Finally, other residual confounders that are challenging to detect, evaluate, or poorly measured may influence our conclusions.

## Conclusions

Our findings suggested that there were significant nonlinear relationships between multiple IR-related indexes and the risk of CKD. TyG-WHtR had the potential to indicate both the risk of CKD occurrence and the all-cause mortality of CKD patients.

### Supplementary Information

Supplementary Material 1.

## Data Availability

Publicly available datasets were analyzed in the present study. All detailed data can be found here: www.cdc.gov/nchs/nhanes/.

## Abbreviations

CKD: Chronic kidney disease
IR: Insulin resistance
NHANES: National Health and Nutrition Examination Survey
METS-IR: Metabolic score of insulin resistance
HOMA-IR: Homeostatic model assessment for insulin resistance
TyG: Triglyceride glucose index
TyG-WHtR: Triglyceride glucose-waist-to-height ratio
TyG-BMI: Triglyceride glucose-body mass index
CVD: Cardiovascular disease
HEC: Hyperinsulinemic-euglycemic clamp
RCS: Restricted cubic spline
ROC: Receiver operating characteristic
AUC: Area under the curve
OR: Odd ratio
HR: Hazard ratio
CI: Confidence interval
ESKD: End-stage kidney disease
DM: Diabetes mellitus
eGFR: Estimated glomerular filtration rate
UACR: Urinary albumin-to-creatinine ratio
MetS: Metabolic syndrome
HDL-C: High-density lipoprotein cholesterol
FPG: Fasting plasma glucose
TG: Triglyceride
BMI: Body mass index
SBP: Systolic blood pressure
DBP: Diastolic blood pressure
OGTT: Oral glucose tolerance test
HbA1c: Hemoglobin A1c
PIR: Poverty to income ratio
SD: Standard deviation
US: United States
