# Supplementary material for: The U-shape relationship between insulin resistance-related indexes and chronic kidney disease: a retrospective cohort study from National Health and Nutrition Examination Survey 2007–2016
Source: Diabetol Metab Syndr. 2024 Jul 17;16:168. doi: 10.1186/s13098-024-01408-7 (PMC11253359; doi:10.1186/s13098-024-01408-7)
Supplement: Supplementary file 1 — Supplementary Material 1. [file 13098_2024_1408_MOESM1_ESM.docx]

Supplementary Material

**Supplementary Table 1.** Association of metabolic score of insulin resistance (METS-IR) with all-cause and cardiovascular disease (CVD) mortality in chronic kidney disease patients

|  | METS-IR  HR^a^ (95% CI^b^) | *P* value |
| --- | --- | --- |
| All-cause mortality | | |
| Model 1^c^ | 0.99  (0.99, 1.00) | 0.047 |
| Model 2^d^ | 1.00  (0.99, 1.00) | 0.323 |
| Model 3^e^ | 1.00  (0.99, 1.02) | 0.329 |
| CVD mortality | | |
| Model 1^c^ | 1.00  (0.99, 1.02) | 0.723 |
| Model 2^d^ | 1.00  (0.99, 1.02) | 0.566 |
| Model 3^e^ | 1.00  (0.97, 1.02) | 0.884 |

^a^ HR: hazard ratio.

^b^ 95% CI: 95% confidence interval.

^c^ Model 1 was unadjusted.

^d^ Model 2 was adjusted for age, gender, race, education level, marital status, and poverty to income ratio.

^e^ Model 3 includes adjustment for variables in model 2 plus body mass index, smoking status, alcohol status, hypertension, abnormal glucose metabolism, hyperlipidemia, and hyperuricemia.

**Supplementary Table 2.** Association of homeostatic model assessment for insulin resistance (HOMA-IR) with all-cause and cardiovascular disease (CVD) mortality in chronic kidney disease patients

|  | HOMA-IR  HR^a^ (95% CI^b^) | *P* value |
| --- | --- | --- |
| All-cause mortality | | |
| Model 1^c^ | 1.00  (1.00, 1.01) | 0.296 |
| Model 2^d^ | 1.01  (1.00, 1.01) | 0.131 |
| Model 3^e^ | 1.01  (1.00, 1.02) | 0.091 |
| CVD mortality | | |
| Model 1^c^ | 1.00  (0.98, 1.02) | 0.934 |
| Model 2^d^ | 1.00  (0.98, 1.02) | 0.987 |
| Model 3^e^ | 1.00  (0.98, 1.02) | 0.844 |

^a^ HR: hazard ratio.

^b^ 95% CI: 95% confidence interval.

^c^ Model 1 was unadjusted.

^d^ Model 2 was adjusted for age, gender, race, education level, marital status, and poverty to income ratio.

^e^ Model 3 includes adjustment for variables in model 2 plus body mass index, smoking status, alcohol status, hypertension, abnormal glucose metabolism, hyperlipidemia, and hyperuricemia.

**Supplementary Table 3.** Association of triglyceride glucose index (TyG) with all-cause and cardiovascular disease (CVD) mortality in chronic kidney disease patients

|  | TyG  HR^a^ (95% CI^b^) | *P* value |
| --- | --- | --- |
| All-cause mortality | | |
| Model 1^c^ | 1.14  (1.01, 1.28) | 0.041 |
| Model 2^d^ | 1.14  (0.99, 1.31) | 0.075 |
| Model 3^e^ | 1.11  (0.93, 1.32) | 0.251 |
| CVD mortality | | |
| Model 1^c^ | 0.85  (0.66, 1.09) | 0.204 |
| Model 2^d^ | 0.86  (0.65, 1.10) | 0.210 |
| Model 3^e^ | 0.89  (0.65, 1.21) | 0.463 |

^a^ HR: hazard ratio.

^b^ 95% CI: 95% confidence interval.

^c^ Model 1 was unadjusted.

^d^ Model 2 was adjusted for age, gender, race, education level, marital status, and poverty to income ratio.

^e^ Model 3 includes adjustment for variables in model 2 plus body mass index, smoking status, alcohol status, hypertension, abnormal glucose metabolism, hyperlipidemia, and hyperuricemia.

**Supplementary Table 4.** Association of triglyceride glucose-body mass index (TyG-BMI) with all-cause and cardiovascular disease (CVD) mortality in chronic kidney disease patients

|  | TyG-BMI  HR^a^ (95% CI^b^) | *P* value |
| --- | --- | --- |
| All-cause mortality | | |
| Model 1^c^ | 1.00  (1.00, 1.00) | 0.048 |
| Model 2^d^ | 1.00  (1.00, 1.00) | 0.285 |
| Model 3^e^ | 1.00  (1.00, 1.00) | 0.350 |
| CVD mortality | | |
| Model 1^c^ | 1.00  (1.00, 1.00) | 0.822 |
| Model 2^d^ | 1.00  (1.00, 1.00) | 0.690 |
| Model 3^e^ | 0.99  (1.00, 1.00) | 0.588 |

^a^ HR: hazard ratio.

^b^ 95% CI: 95% confidence interval.

^c^ Model 1 was unadjusted.

^d^ Model 2 was adjusted for age, gender, race, education level, marital status, and poverty to income ratio.

^e^ Model 3 includes adjustment for variables in model 2 plus body mass index, smoking status, alcohol status, hypertension, abnormal glucose metabolism, hyperlipidemia, and hyperuricemia.
